# Hypoxia/HIF1α induces lapatinib resistance in ERBB2-positive breast cancer cells via regulation of DUSP2

**DOI:** 10.18632/oncotarget.2806

**Published:** 2015-01-24

**Authors:** Sergey V. Karakashev, Mauricio J. Reginato

**Affiliations:** ^1^ Department of Biochemistry and Molecular Biology, Drexel University College of Medicine, Philadelphia, PA 19102, USA

**Keywords:** breast cancer, hypoxia, HIF-1α, lapatinib, ERBB2/HER2, DUSP2

## Abstract

ERBB2/HER2 belongs to the EGFR-family of receptor tyrosine kinases and its overexpression can promote tumor progression. Breast cancer patients with ERBB2 amplifications are currently treated with lapatinib, a small-molecule kinase inhibitor that specifically blocks EGFR/ERBB2 signaling. Here, we show that hypoxia, via HIF-1, induces resistance to lapatinib-mediated effects in ERBB2-expressing mammary epithelial and ERBB2-positive breast cancer cells. Lapatinib-mediated growth inhibition and apoptosis in three-dimensional (3D) cultures are decreased under hypoxic conditions. Hypoxia can maintain activation of signaling pathways downstream from ERBB2 including AKT and ERK in the presence of lapatinib. HIF-1 is both required and sufficient to induce lapatinib resistance as overexpression of stable HIF-1 in ERBB2-expressing cells blocks lapatinib-mediated effects and maintains ERBB2-downstream signaling under normoxic conditions. Under hypoxia, activation of ERK signaling is required for lapatinib resistance as treatment with MEK inhibitor trametinib reverses hypoxia-mediated lapatinib resistance. HIF-1 can bypass the lapatinib-treated inhibition of the ERK pathway via inhibition of the dual-specificity phosphatase 2 (DUSP2). Indeed, overexpression of DUSP2 in ErbB2-positve breast cancer cells reverses hypoxia-mediated lapatinib resistance. Thus, our results provide rationale for therapeutic evaluation of the treatment of hypoxic ERBB2 expressing breast tumors with a combination of lapatinib and MEK inhibitors.

## INTRODUCTION

Despite significant progress in treatment and diagnostics, breast cancer remains the second most deadly cancer among women in the developed world [1]. Breast cancer is a highly heterogeneous disease, classified by stage, size, morphology and the presence of receptors such as estrogen receptor, progesterone receptor or ERBB2/HER2. ERBB2 is a receptor tyrosine kinase that is overexpressed in over 30% of breast tumors [2, 3]. This oncogene belongs to the EGFR receptor family and plays an important role in EGFR pathway signaling. This pathway is triggered by EGFR activation via binding to its ligand EGF resulting in activation of EGFR/ERBB2 downstream target proteins including the serine/threonine kinase AKT and extracellular signal-regulated kinase (ERK). ERK kinase regulates multiple downstream targets involved in regulation of cell proliferation and survival and the ERK pathway is deregulated in many cancers.

Since ERBB2 overexpression in breast cancer cells is critical for tumor progression it is an attractive therapeutic target [4]. Indeed, development of targeted therapy against EGFR/ERBB2, such as a lapatinib, have significantly improved treatment of ERBB2-positive breast cancer [5, 6]. Lapatinib, a small molecule kinase inhibitor, blocks both EGFR and ERBB2 kinase activity and is currently used in combination therapy with DNA damaging agents in ERBB2-positive breast cancers [7]. In spite of the clinical efficiency of lapatinib in breast cancer patients, some ERBB2-expresing tumors are not responsive to this treatment and some patients that do initially respond, acquire lapatinib resistance [8, 9]. Understanding molecular mechanisms of lapatinib-resistance may help identify combination regimens for these patients.

Hypoxia, or low oxygen concentration, is another factor that contributes to tumor progression. Normal epithelial cells usually have 3–7% oxygen tension. However, in solid tumors, oxygen concentrations often reach below 1% due to uncontrolled cell growth and the inability to form normal blood vessels [10]. Hypoxia inducible factor 1 (HIF-1) is a major player in the cells response to hypoxia. HIF-1 is a transcription factor that consists of two subunits: HIF-1α and HIF-1β. Under normoxic conditions, HIF-1α is hydroxylated by prolyl hydroxylase (PHD) that utilizes oxygen as a substrate and hydroxylated HIF-1α undergoes rapid proteasomal degradation [11]. However, at low oxygen levels PHD is not able to hydroxylate HIF-1α and it translocates to the nucleus where it forms a dimer with HIF-1β. HIF-1 dimer activates expression of more than one hundred genes that play a protective role in cells experiencing hypoxia [12, 13]. Many of HIF-1α-mediated effects are beneficial for transformed cells as HIF-1α reprograms cell metabolism towards glycolysis [14], activates angiogenesis [15], and inhibits apoptosis [16]. Therefore, hypoxic tumors are often associated with poor prognosis and are resistant to different anticancer agents [17–19]. However, the mechanism of hypoxia/HIF-1-mediated resistance to targeted therapy has not been well studied.

Here, we show that hypoxia promotes lapatinib resistance in ERBB2- expressing breast cancer cells through HIF-1-mediated ERK activation. Specifically, HIF-1α stabilization in hypoxic cells leads to activation of ERK pathway via downregulation of dual-specificity phosphatase 2 (DUSP2). Indeed, the expression of a stable HIF-1α mutant or reduction of DUSP2 via RNAi can promote lapatinib resistance in breast cancer cells under normal oxygen tension. In addition, we show that targeting the ERK pathway in hypoxic ERBB2-positive breast cancer cells sensitizes to lapatinib treatment. Importantly, HIF-1α expression inversely correlates with DUSP2 levels in ER-negative breast cancer patients and associates with poor prognosis.

## RESULTS

### Hypoxia blocks lapatinib-mediated growth inhibition in ERBB2-positive breast cancer cells

**Figure 1:**
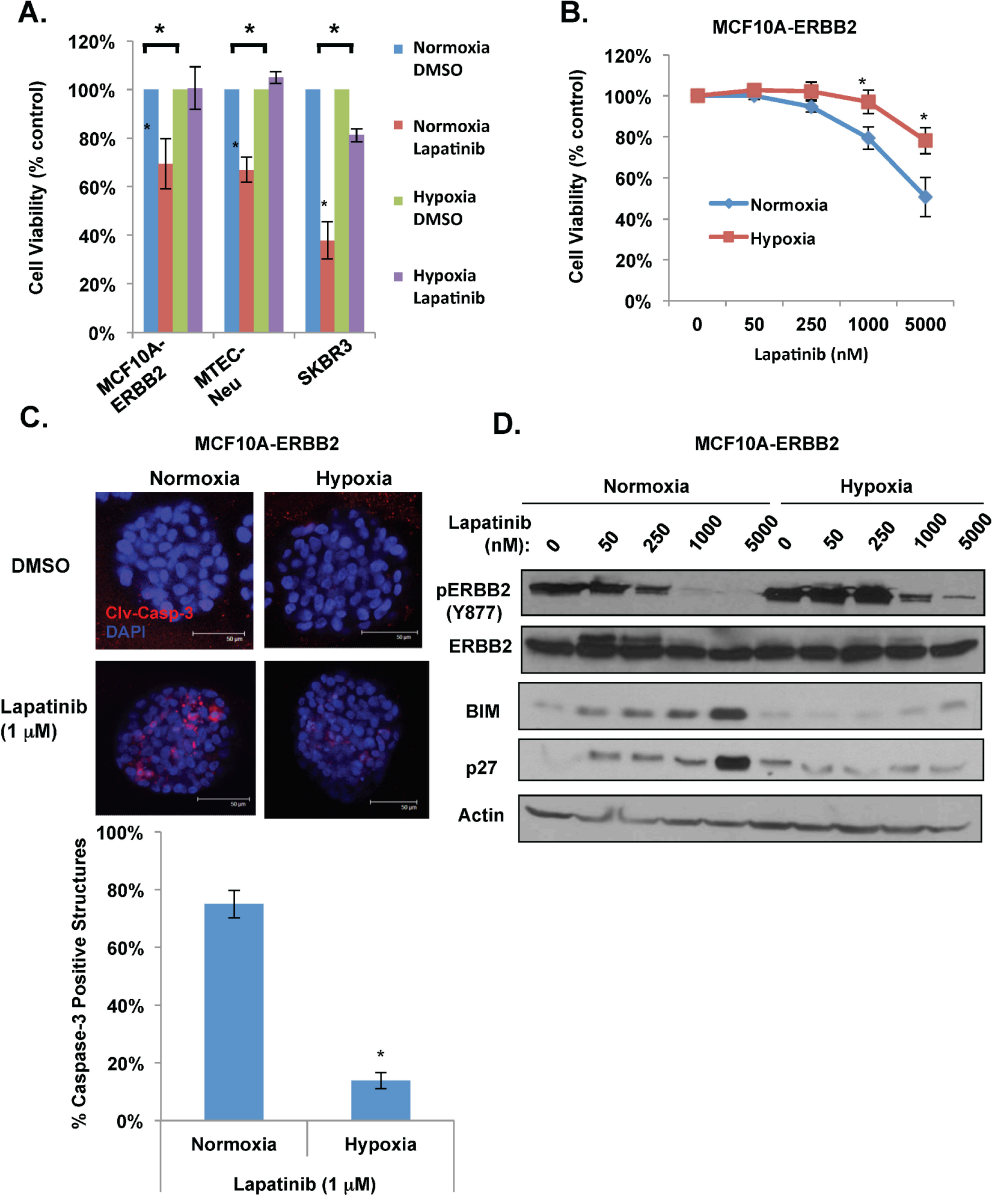
Hypoxia blocks lapatinib-mediated effects in ERBB2-positive breast cancer cells **(A)** Indicated cells were treated with 1 μM lapatinib under hypoxia for 48h and cell viability was assessed by MTS assay. **(B)** MCF10A-ERBB2 cells were treated with increasing doses of lapatinib under normoxic or hypoxic conditions and cell viability was assessed. **(C)** Cell were placed in 3D culture conditions and transferred to normoxic or hypoxic conditions in the presence or absence of lapatinib. Cells were then stained for cleaved caspase-3 (top) and the percentage of caspase-positive acini was determined (bottom). **(D)** Cell lysates were collected from cells in B for immunoblot analysis. Error bars indicate S.E. (*p ≤ 0.05).

We have previously shown that treatment of MCF-10A cells with hypoxia (1% O2) blocks anoikis and inhibits luminal clearing of acinar-like structures when cells are placed in three dimensional (3D) basement membrane cultures [20]. More recently, we showed that MCF-10A cells overexpressing ERBB2 and ERBB2-positive breast cancer cells stabilizes HIF-1α levels and that HIF-1α is required for ERBB2 oncogenesis *in vivo* and anoikis resistance *in vitro* [21]. Since hypoxia is associated with resistance to standard chemotherapy [22], we examined whether hypoxia alters response of ERBB2-positive breast cancer cells to targeted therapies such as lapatinib. Using MCF10A cells overexpressing wild type ERBB2 (MCF10A-ERBB2), mammary tumor epithelial cells derived from MMTV-*neu* transgenic mice (MTEC-Neu) and SK-BR3 cells, all of which overexpress similar levels of ERBB2 (Figure S1A), we examined the effects of lapatinib treatment under normoxic and hypoxic (1% O2) conditions. Treatment of all three cell lines with lapatinib (1 μM) under normoxic conditions reduced cell viability as measured by MTS assay (Figure 1A). However, under hypoxic conditions, treatment with lapatinib had reduced effects on cell viability in MCF10A-ERBB2, MTEC-Neu and SK-BR3 cells (Figure 1A).

To characterize this effect further, we examined MCF10A-ERBB2 cells treated with increasing doses of lapatinib for 48 hours under normoxic and hypoxic conditions. Treatment of MCF10A-ERBB2 cells with lapatinib, under normal oxygen conditions, showed a decrease in viability of 21% and 49% at 1 and 5 μM respectively compared to control treated cells (Figure 1B). However, treatment under hypoxic conditions showed a decrease of viability of only 3% and 22% at same doses (Figure 1B). To verify MTS results, we carried out cell counting and observed similar inhibition of lapatinib effects on MCF10A-ERBB2 cell number under hypoxic conditions compared to normoxia (Figure S1B). In order to determine whether hypoxia alters the effects of lapatinib on MCF10A-ERBB2 cells cultured in 3D conditions, single MCF-10A-ERBB2 cells were placed in basement membrane culture as previously described [23] and allowed to form acinar-like structures for six days under normal oxygen. Cells were then treated with 1 μM lapatinib and either maintained in normoxic conditions or placed in hypoxic conditions for 48h. Lapatinib treatment of ERBB2 cells under normoxic conditions contained 75% cleaved-caspase-3 positive structures (Figure 1C). However, hypoxia-treated structures contained 5 fold less caspase-3 cleavage (14%) following lapatinib treatment. Thus, hypoxia blocks lapatinib-mediated cell death in ERBB2-positive breast cancer cells in both standard and in 3D culture conditions.

We next examined if hypoxia alters lapatinib effects on ERBB2-mediated signaling. As expected, MCF10A-ERBB2 cells treated with lapatinib for 48 hours under normoxic conditions contained decreased ERBB2 phosphorylation (Y877) starting at 250 nM concentration and maximally inhibited ERBB2 phosphorylation at 1 and 5 μM (Figure 1D). However, under hypoxia we observed that lapatinib treated cells maintained ERBB2 activation and ERBB2 remained active at 1 and 5 μM treatments compared to normoxic cells (Figure 1D). We also examined expression of the Bcl-2-family pro-apoptotic protein BIM and cell cycle inhibitor p27^Kip1^. These two proteins are downstream of ERBB2/EGFR pathway and are often used as biomarkers for efficiency of anti-ERBB2 therapy [24–26]. Expression of both BIM and p27^Kip1^ were upregulated in normoxic cell treated with higher lapatinib doses (Figure 1D). However, consistent with hypoxia blocking lapatinib-effects on apoptosis in 3D conditions and cell growth in 2D, hypoxia prevented lapatinib-mediated increase in expression of both BIM and p27^Kip1^ levels (Figure 1D). Thus, hypoxia can reduce lapatinib-mediated inhibition of ERBB2 phosphorylation and induction of key regulators of apoptosis and cell cycle arrest in ERBB2-expressing cells.

### ERK activity in elevated and required for hypoxia-mediated lapatinib resistance in breast cancer cells

**Figure 2:**
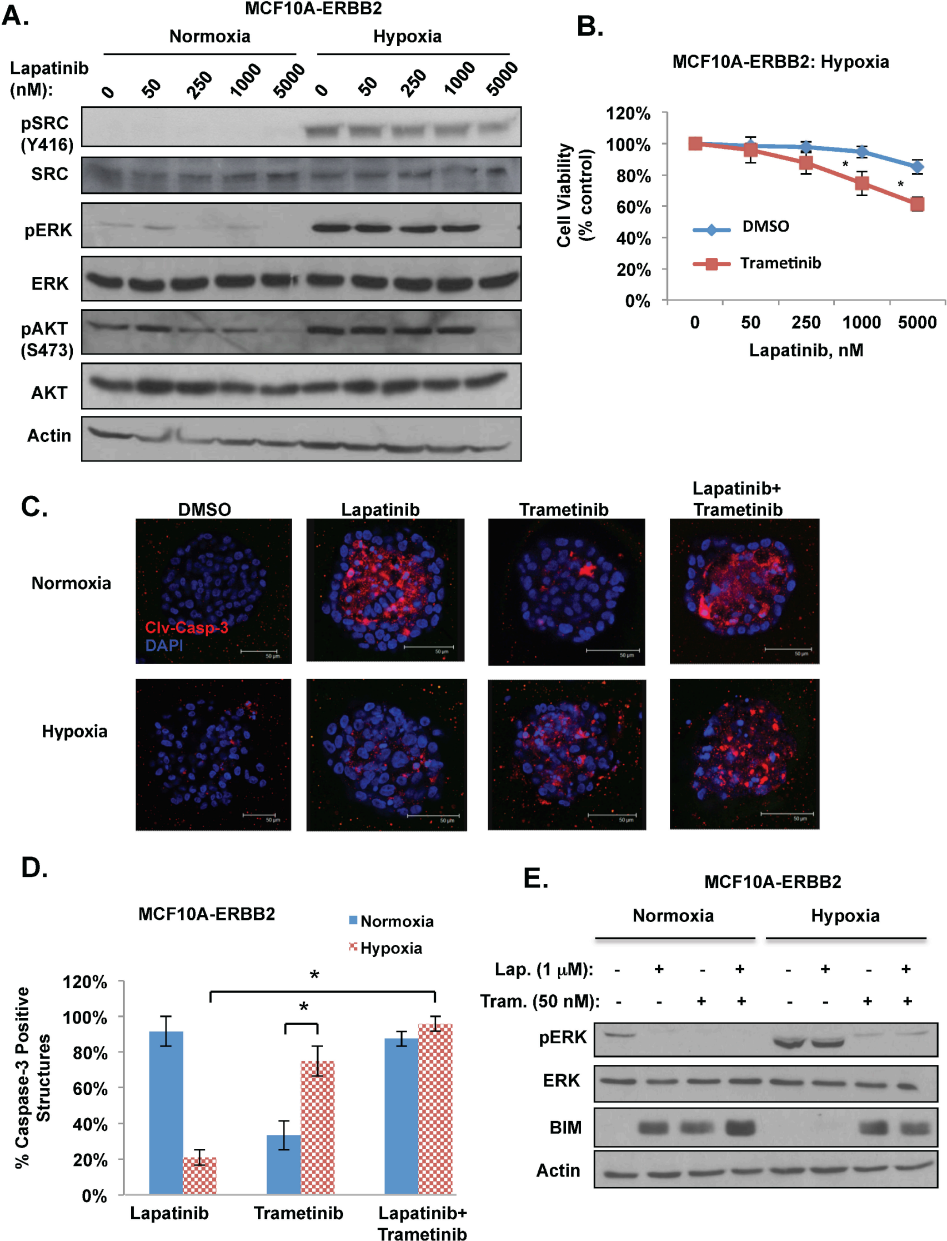
Hypoxia requires ERK activity for lapatinib resistance in breast cancer cells **(A)** Cells were treated with increasing doses of lapatinib under normoxic or hypoxic conditions and cell lysates were collected for immunoblot analysis. **(B)** Cells were treated with increasing doses of lapatinib under hypoxic conditions in the presence of control or trametinib (50 nM) and cell viability was assessed. **(C)** MCF10A-ERBB2 cells were placed in 3D culture conditions and then incubated under normoxic or hypoxic conditions in the presence or absence of lapatinib (1 μM), trametinib (50 nM) or both treatments. Cells were stained for cleaved caspase-3. **(D)** The percentage of caspase-positive acini was determined for C. **(E)** Cells were treated with lapatinib, trametinib or a combination of both drugs under normoxic or hypoxic conditions and cell lysates were collected for immunoblot analysis. Error bars indicate S.E. (*p ≤ 0.05).

We next examined ERBB2 downstream signaling in lapatinib treated MCF10A-ERBB2 cells in response to hypoxia. As expected, lapatinib treatment of ERBB2-expressing cells reduced ERK and AKT activation under normoxic conditions (Figure 2A). However, under hypoxia ERK and AKT activation was increased and maintained even in presence of high doses of lapatinib (Figure 2A). Since recent studies have shown that hypoxia can also activate c-SRC [27], we examined c-SRC activation under these conditions. We found undetectable levels of c-SRC activation in normoxic cells. However, c-SRC is strongly activated in cells exposed to hypoxia and lapatinib treatment has minimal effects on c-SRC activation in these cells (Figure 2A). Thus, hypoxia increases and prolongs c-SRC, ERK and AKT activation in ERBB2-expressing cells in the presence of lapatinib.

Activation of c-SRC, AKT and ERK pathways has been shown previously to play a role in resistance to ERBB2-targeted therapy [28] thus we next determined which pathway is critical for hypoxia mediated lapatinib resistance. MCF10A-ERBB2 cells were treated with either phosphoinositide 3-kinase (PI3K) inhibitor (LY294002) or MEK inhibitor (UO126) in the presence of increasing doses of lapatinib. Under normoxic conditions, treating cells with PI3 kinase or MEK inhibitor did not alter lapatinib effects on cell viability (Figure S2A). However, under hypoxic conditions, MEK inhibitor, but not PI3K inhibitor, reduced cell viability in lapatinib treated cells (Figure S2A). Since we also observed increased levels of c-SRC phosphorylation in MCF10A-ERBB2 cells under hypoxic conditions (Figure 2A), we tested whether c-SRC activation is required for hypoxia-mediated lapatinib resistance. MCF10A-ERBB2 cells under hypoxia were treated with vehicle control or dasatinib, a small molecule c-SRC inhibitor. Treatment of MCF10A-ERBB2 cells with dasatinib inhibited hypoxia-mediated c-SRC activation (Figure S3A) but did not alter hypoxia-mediated effects on growth in lapatinib treated cells (Figure S3B). Moreover, dasatinib treatment did not alter ERK activation under hypoxic or normoxic conditions (Figure S3A). Thus, hypoxia-mediated lapatinib resistance in MCF10A-ERBB2 cells is dependent on MEK/ERK activity and is independent of AKT and c-SRC pathways.

### Trametinib abrogates hypoxia-mediated lapatinib resistance in ERBB2 breast cancer cells

Recently, a novel MEK inhibitor trametinib has been approved for treatment of metastatic melanoma containing B-RAF mutations as monotherapy or in combination with dabrafenib [29]. Thus, we tested whether trametinib, a selective MEK inhibitor, can decrease the protective effect hypoxia provides to lapatinib treated ERBB2 expressing cells. MCF10A-ERBB2 cells were treated with trametinib in the presence of increasing doses of lapatinib under normoxic and hypoxic conditions. Treatment with trametinib did not alter lapatinib-mediated effects on cell viability under normoxic conditions (Figure S2B). However, under hypoxic conditions, the addition of trametinib reduced cell viability in lapatinib treated cells (Figure 2B). Importantly, trametinib treatment abrogated the hypoxia-mediated protective effect on lapatinib treated 3D structures. Combination treatment of lapatinib with trametinib reversed hypoxic-mediated inhibition of apoptosis in acinar structure that was observed when treated with lapatinib alone (Figure 2C and 2D). Interestingly, trametinib treatment alone in 3D structures had much stronger apoptotic effect on hypoxic cells compared to normoxic cells (Figure 2C and 2D). In addition, treating MCF10A-ERBB2 cells with trametinib alone or in combination with lapatinib reversed hypoxia-mediated activation of ERK and inhibition of BIM expression (Figure 2E). Similar results were seen in hypoxic breast cancer cells derived from MMTV-Neu mice (MTEC-Neu) cultured in 3D and treated with lapatinib and trametinib (Figure S4). Thus, these data suggest that combination treatment of lapatinib and trametinib should be explored as a therapeutic option in treating hypoxic ERBB2-positive breast cancer cells.

### HIF-1α is required for hypoxic effects on proliferation and signaling in lapatinib-treated ERBB2-positive cells

**Figure 3:**
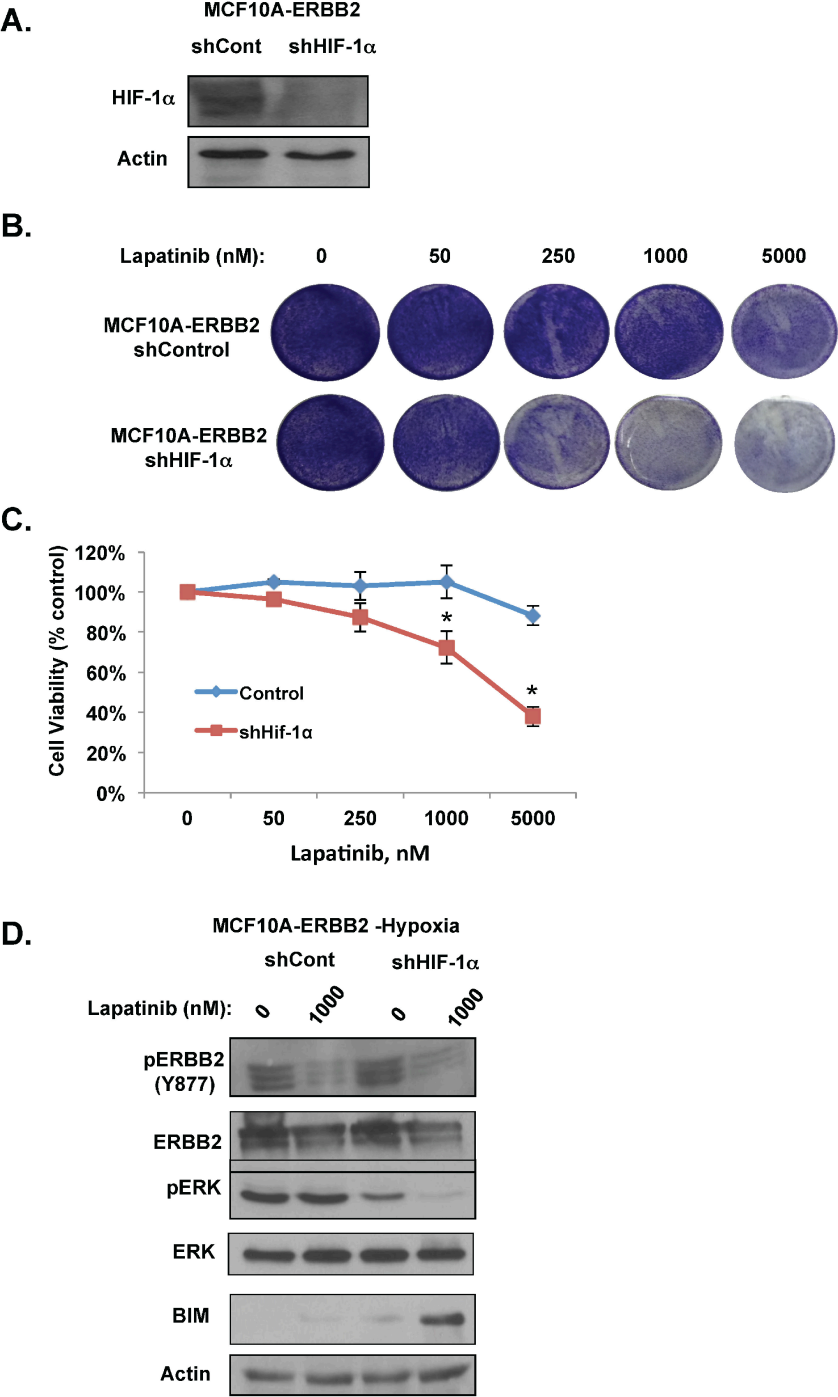
HIF-1α is required for hypoxic-mediated lapatinib resistance and signaling in ERBB2-expressing cells **(A)** Cell lysates from cells stably expressing control or HIF-1α shRNA were collected for immunoblot analysis. **(B)** Cells were treated with increasing doses of lapatinib under hypoxic conditions and then stained with crystal violet. **(C)** Cell viability was assessed with cells as described in B. **(D)** Cells expressing control or HIF-1α shRNA were treated with vehicle control or lapatinib under hypoxic conditions. Cell lysates were then collected for immunoblot analysis. Error bars indicate S.E. (*p ≤ 0.05).

To investigate how hypoxia activates ERK signaling and leads to lapatinib resistance in MCF10A-ERBB2 cells, we first examined the role of HIF-1. Since HIF-1α stabilization and induction of a number of genes are major events in cellular responses to hypoxia [30], we examined whether HIF-1 is required for hypoxia-mediated lapatinib resistance in MCF10A-ERBB2 cells. We stably reduced HIF-1α levels in MCF10A-ERBB2 cells via shRNA (Figure 3A). Reducing HIF-1α levels in MCF10A-ERBB2 cells had minimal effects under hypoxic conditions in 2D culture; however HIF-1α depleted cells were sensitized to lapatinib treatment starting at 250 nM dose as seen by crystal violet staining (Figure 3B). Hypoxic cells stably expressing HIF-1α shRNA were no longer resistant to lapatinib treatment (Figure 3C). We also examined effects of reducing HIF-1α on signaling pathways and biomarkers in lapatinib treated cells. Reducing HIF-1α had no effect on ERBB2 phosphorylation in untreated or lapatinib-treated cells under hypoxic conditions (Figure 3D). As shown above, hypoxia maintained ERK activation in lapatinib treated cells (Figure 3D). However, reducing HIF-1α levels in MCF10A-ERBB2 hypoxic cells reduced basal ERK activity and importantly restored lapatinib-mediated ERK inhibition. HIF-1α knockdown cells treated with lapatinib also contained elevated BIM levels compared to control cells (Figure 3D), consistent with reduced cell viability of these cells under hypoxia. Thus, these data suggest that HIF-1α is required for hypoxia-mediated resistance to lapatinib effects, activation of ERK and BIM inhibition.

### HIF-1 is sufficient to induce resistance to lapatinib in ERBB2-positive cells under normoxic conditions

**Figure 4:**
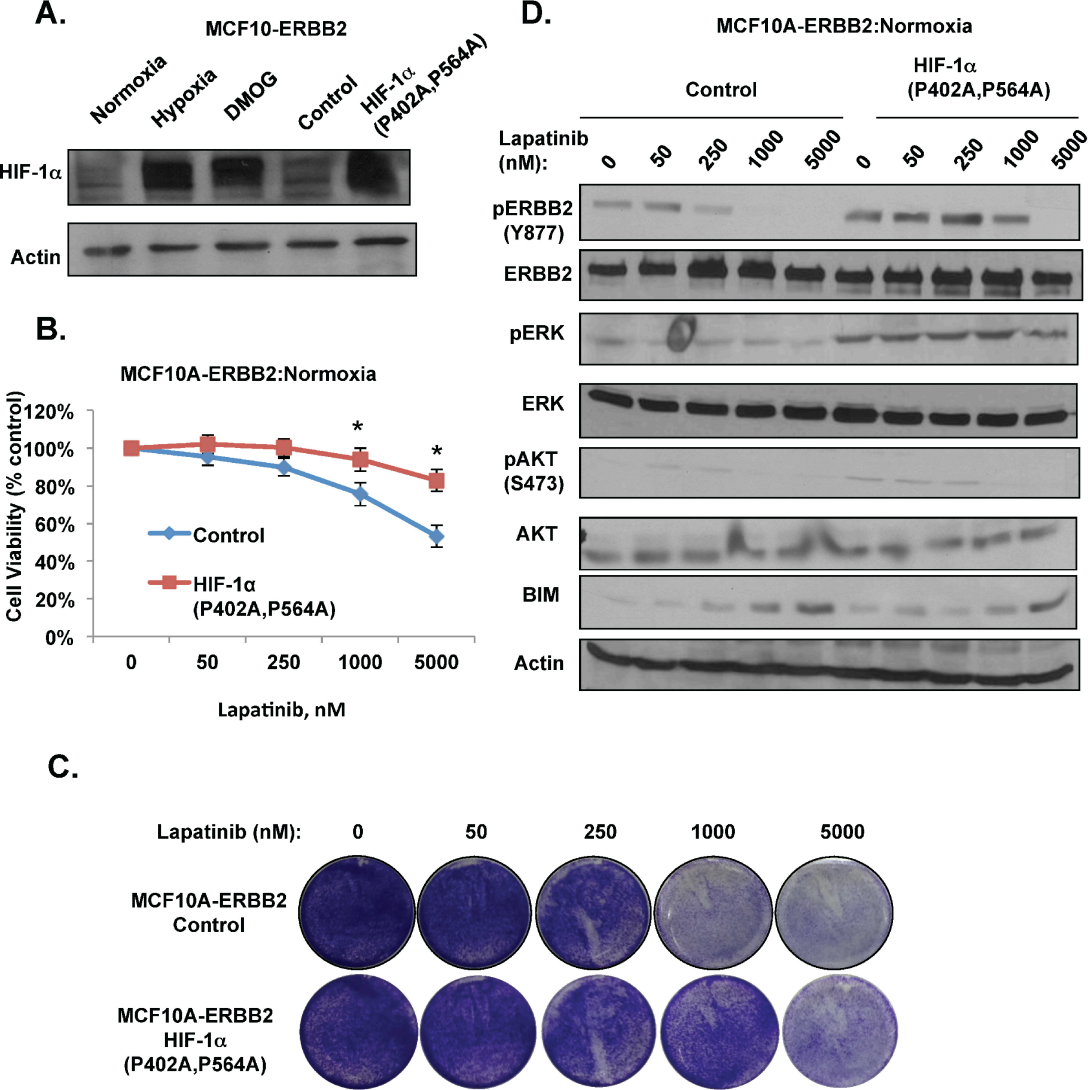
HIF-1α is sufficient to induce lapatinib-resistance in ERBB2-expressing cells under normoxic conditions **(A)** Cell lysates from cells placed in hypoxia, treated with DMOG for 6 hrs, or cells expressing control or HIF-1α mutant (P402A/P564A) were collected for immunoblot analysis. **(B)** MCF10A-ERBB2 cells stably expressing control or HIF-1α mutant were treated with increasing doses of lapatinib and cell viability was assessed. **(C)** Cells as in B were stained with crystal violet. **(D)** Cell lysates were collected from cells as in B for immunoblot analysis. Error bars indicate S.E. (*p ≤ 0.05).

To determine whether HIF-1α stabilization is sufficient to confer lapatinib resistance in ERBB2-expressing cells under normoxic conditions, we overexpressed a stable non-degradable form of HIF-1α containing proline to alanine mutations (HIF-1α P402A, P564A) in MCF10A-ERBB2 cells. We confirmed that this mutant expressed levels similar to endogenous HIF-1α stabilized under hypoxia and to cells treated with prolyl-hydroxylase inhibitor DMOG (Figure 4A). MCF10A-ERBB2 cells expressing the stable form of HIF-1α were resistant to lapatinib-mediated effects on cell viability (Figure 4B) and cell number (Figure 4C) under normal oxygen conditions. In addition, similar to effect of hypoxia, MCF10A-ERBB2 cells expressing stable form of HIF-1α were able to maintain ERBB2, ERK, and AKT activation even when treated with high doses of lapatinib under normal oxygen conditions (Figure 4D). Stable HIF-1α expressing cells also contained reduced levels of BIM following lapatinib treatment compared to control cells (Figure 4D). Thus, HIF-1α expression alone is sufficient to block lapatinib-mediated effect on growth and signaling in ERBB2-expressing cells under normal oxygen conditions.

### Hypoxia/HIF-1promotes lapatinib resistance through regulation of DUSP2

**Figure 5:**
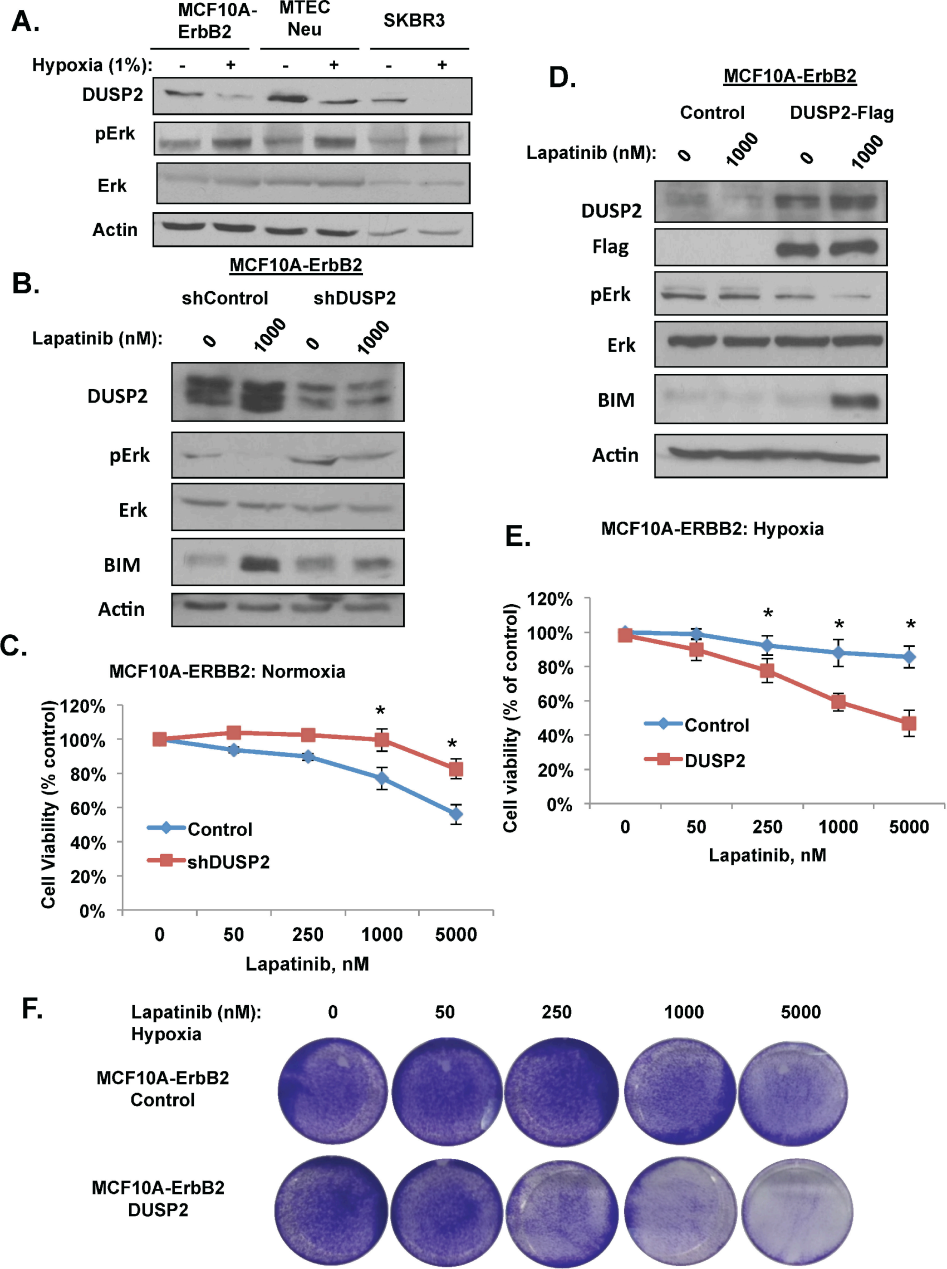
HIF-1 target DUSP2 is required for lapatinib resistance **(A)** Indicated cells were exposed to hypoxia for 6 hrs and cell lysates were collected for immunoblot analysis. **(B)** Cells stably expressing control or DUSP2 shRNA were treated with 1 μM lapatinib and cell lysates were collected for immunoblot analysis. **(C)** Cells expressing control or DUSP2 shRNA were treated with increasing doses of lapatinib and cell viability was assessed. **(D)** Cells stably expressing DUSP2-flag protein were treated with 1 μM lapatinib under hypoxia and cell lysates were collected for immunoblot analysis. **(E)** Cells expressing DUSP2-flag were treated with increasing doses of lapatinib and cell viability was assessed. **(F)** Cells as in E were stained with crystal violet. Error bars indicate S.E. (*p ≤ 0.05).

One possible mechanism of HIF1-mediated ERK activation under hypoxia is regulation of the dual specificity protein phosphatase 2 (DUSP2). DUSP2 is a phosphatase that negatively regulates ERK activity [31]. Recently, it has been reported that DUSP2 is downregulated in many cancers and that hypoxic tumors have decrease expression of DUSP2 [32]. We examined DUSP2 expression in three different ERBB2 expressing breast cancer cell lines and observed that DUSP2 protein levels are downregulated under hypoxic conditions (Figure 5A). DUSP2 RNA levels were also reduced nearly 90% in hypoxic MCF10-ERBB2 when compared to normal oxygen (Figure S5A). Consistent with its regulation of ERK, DUSP2 downregulation correlated with increased ERK activation in breast cancer cells placed under hypoxic conditions (Figure 5A). Moreover, MCF10A-ERBB2 cells expressing HIF-1α RNAi abrogated DUSP2 downregulation under hypoxia, (Figure S5B) thus regulation of DUSP2 by hypoxia in breast cancer cells is HIF-1α-dependent.

To test whether DUSP2 inhibition alone was sufficient to confer lapatinib resistance in ERBB2-expressing cells under normal oxygen conditions, we stably reduced DUSP2 expression in MCF10A-ERBB2 cells via RNAi. As expected DUSP2 inhibition resulted in increased ERK activation even under normoxic conditions (Figure 5B). MCF10A-ERBB2 cells depleted of DUSP2 partially blocked lapatinib effect on cell viability under normoxic conditions (Figure 5C and S6A). We also tested if DUSP2 depletion inhibits the effects lapatinib has on MCF10A-ERBB2 cells cultured in 3D conditions. Control and DUSP2 depleted cells were cultured in 3D for 6 days under normoxic conditions and then treated with lapatinib for 48 hrs. DUSP2 downregulation alone was sufficient to significantly reduce caspase-3 cleavage in lapatinib treated cells (Figure S6B). Similar results were seen in DUSP2 depleted SK-BR3 cells with respect to ERK activation (Figure S6C) and inhibition of lapatinib-mediated effects on cell viability under normoxic conditions (Figure S6D). These data suggest that reducing DUSP2 levels alone in ERBB2 expressing breast cancer cells induces lapatinib resistance. To test whether increasing DUSP2 levels could sensitize hypoxic breast cancer cells to lapatinib, we stably overexpressed Flag-tagged DUSP2 in MCF10A-ErbB2 cells. Compared to control cells, cells overexpressing DUSP2 were sensitized to lapatinib-treatment as Erk activation was reduced and BIM expression elevated in these cells (Figure 5D). DUSP2 overexpressing cells reversed hypoxia-mediated resistance to lapatinib treatment as measured by cell viability (Figure 5E) and cell number (Figure 5F). These data suggest that hypoxia-mediated lapatinib resistance in ErbB2-positive breast cancer cells is DUSP2 dependent.

### Inverse DUSP2 and HIF-1α relationship in ER negative breast cancers is associated with poor prognosis

**Figure 6:**
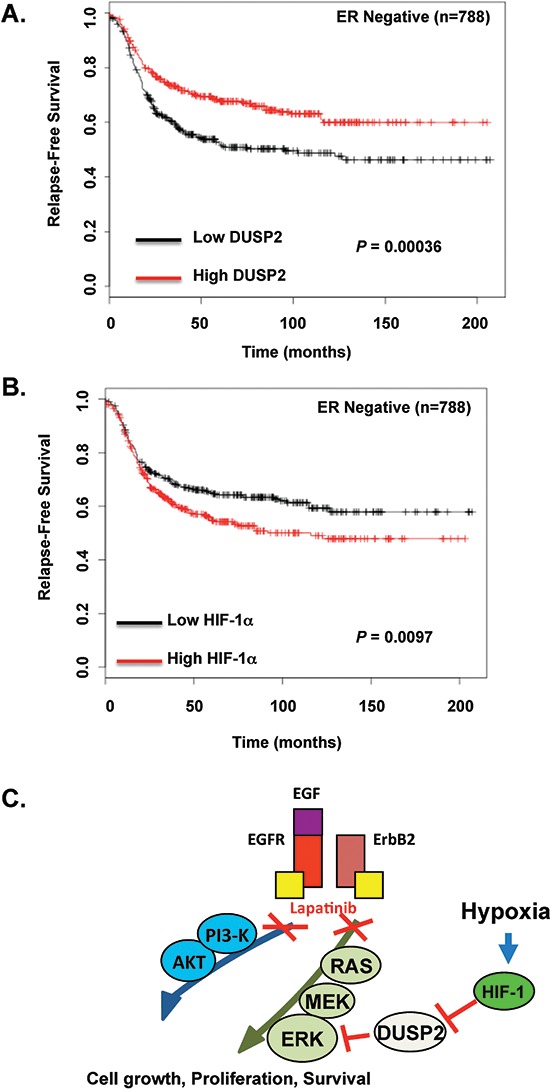
An inverse relationship between DUSP2 and HIF-1α in ER negative breast cancer associates with decreased relapse-free survival **(A)** Kaplan-Meier plots of relapse-free survival (RFS) in a dataset of patients with ER-negative breast cancer (*n* = 788), stratified by DUSP2 expression. Data was obtained from the Kaplan-Meier plotter database [34]. The *P* value was calculated by a log-rank test. **(B)** Similar analysis was performed in the same patient database but RFS stratified by HIF-1α expression. **(C)** A schematic model of hypoxia-mediated lapatinib resistance.

Since HIF-1α expression is associated with poor clinical outcome in breast cancer patients [33] and we show that hypoxia via HIF-1α reduces DUSP2 expression, we interrogated whether DUSP2 expression may also be associate with poor clinical outcome in breast cancer patients. We examined expression of DUSP2 in ERBB2-positive breast cancer samples using Kaplan-Meier Plotter [34] and found that in 207 ERBB2-positive breast cancer patients with reduced DUSP2 levels showed a decreased trend in relapse-free survival that was not significant (*p* = 0.15) (Figure S7). However, when we examined a larger cohort of ER-negative breast cancer patients (*n* = 788 patients) we found that low levels of DUSP2 expression was significantly associated with reduced relapse-free survival (Figure 6A). In addition, in this same patient population we found that high levels of HIF-1α is also associated with reduced relapse-free survival (Figure 6B), which is consistent with the idea that HIF-1α reduces DUSP2 expression and thus may inversely correlate with DUSP2 levels. Thus, an inverse relationship exists between DUSP2 and HIF-1α in ER negative breast cancer patients with both high HIF-1α and low DUSP2 levels associating with poor prognosis.

## DISCUSSION

Here, we demonstrate for the first time that hypoxia promotes lapatinib resistance in ERBB2-positive breast cancer cells through activation of the MEK-ERK pathway in a HIF-1-dependent manner via regulation of DUSP2 (Figure 6C). Additionally, we show that hypoxia abrogated the induction of apoptosis in 3D cultures treated with lapatinib. We also demonstrate that lapatinib treated ERBB2-positive cell lines exposed to hypoxia maintained high levels of ERK phosphorylation and decreased levels of pro-apoptotic protein BIM compared to lapatinib-treated cells under normal oxygen. Moreover, we show that hypoxia/HIF-1 inhibition of DUSP2 is a major mechanism by which hypoxia activates ERK and inhibits lapatinib-mediated induction of apoptosis and BIM. Importantly, co-treatment with lapatinib and trametinib, an FDA approved MEK inhibitor, was able to reverse hypoxia protective effects. Thus, combination treatment of lapatinib and trametinib may be a therapeutic option in treating hypoxic ERBB2-positive breast cancer cells.

Clinical efficiency of lapatinib is limited by acquired resistance [35] thus understanding mechanisms of resistance may lead to better treatment modalities. Several mechanisms of lapatinib resistance have been proposed, including ERBB2-independent activation of PI3K-AKT and ERK pathways [8, 36]. ERBB2-independent ERK activation has been shown to play a role in lapatinib resistance. In ERBB2/EGFR-positive pancreatic cancer cell lines lapatinib has limited efficacy due to *k-ras* mutations [37]. However, MEK inhibitors can sensitize these tumors to lapatinib [38]. Moreover, SRC, MET, and Axl driven lapatinib-resistant cell lines also have increased ERK signaling. Currently a combination of lapatinib and c-SRC inhibitor as well as lapatinib and MET kinase inhibitor are in clinical trials (https://clinicaltrials.gov NCT00662636 and NCT01138384). Activation of other members of EGFR family such as ERBB1 or ERBB 3 has also been shown to promote lapatinib resistance in various cancer cell lines [39–41]. We and others have shown that hypoxia can activate MEK/ERK pathways and in this study we show for the first time that hypoxia can promote lapatinib resistance through activation of ERK signaling. Indeed, cells exposed to hypoxia had increased c-SRC, AKT and ERK signaling and were less sensitive to lapatinib. Although hypoxia was able to activate multiple pathways in breast cancer cells only treatment with MEK inhibitors were able to reverse the hypoxia-mediated protective effect in lapatinib treated cells suggesting that the ERK pathway is critical for hypoxia-mediated lapatinib resistance. Interestingly, hypoxic ERBB2-expressing cells in 3D culture were hypersensitive to trametinib treatment alone compared to normoxic cells, suggesting that hypoxic cancer cells may be more dependent on the MEK-ERK pathway for cell survival and thus more sensitive to MEK inhibitors.

Recent studies have shown that resistance to targeted therapy may depend on whether tumor cells are in direct contact with extracellular matrix while matrix-deprived cells may be more sensitive to pathway inhibitors [42]. Consistent with this idea, lapatinib treatment of ERBB2-expressing cells in 3D culture under normoxic conditions was less effective at inducing apoptosis in matrix-attached cells compared to inner cells deprived of contact with matrix (Figure 2B). However, under hypoxic conditions we observed reduced amounts of apoptotic cells in both inner and outer regions of 3D structures and, importantly, combination of lapatinib and trametinib was able to induce apoptosis in both matrix-attached and matrix-deprived hypoxic cells. Thus, targeting MEK/ERK pathway in hypoxic tumors may reverse lapatinib resistance from matrix-protection.

To further investigate the mechanism of hypoxia-mediated ERK activation and lapatinib resistance we tested if stabilization of HIF-1α was required for hypoxia mediated ERK activation. In HIF-1 depleted cells hypoxia-mediated lapatinib resistance was reversed as well as hypoxia regulation of ERK phosphorylation and BIM expression. Moreover, expression of a stable form of HIF-1 in ERBB2-expressing cells was sufficient to induce lapatinib resistance, and maintain ERK signaling, even in the presence of high doses of lapatinib under normoxic conditions. Thus, HIF-1 is both required and sufficient for hypoxia-mediated lapatinib-resistance and the effect on cell viability and ERK signaling in ERBB2-positive cells. These data suggest that HIF-1α may be used as a biomarker to predict lapatinib resistance in ERBB2-positive breast cancers.

Lin [32] and others have previously shown that hypoxia/HIF-1 can regulate ERK activity through DUSP2 downregulation. DUSP2 is a phosphatase that negatively regulates ERK and p38 activity. DUSP2 downregulation was previously observed in different types of cancer. In HeLa cells, hypoxia/HIF-1/DUSP2-mediated ERK activation promotes doxycycline and paclitaxel resistance [32]. Interestingly, paclitaxel is widely used in combinational breast cancer therapy including treatment of ERBB2-positive breast tumors. In this study we demonstrate that hypoxia decreases DUSP2 levels in three cancer cell lines and this correlates with increased ERK activation. Moreover, DUSP2 depletion promotes lapatinib resistance even under normoxic conditions and DUSP2 overexpression abrogates hypoxia protective effect on ErbB2-positive cells treated with lapatinib. Data from clinical samples shows that ER-negative breast cancers with reduced DUSP2 expression associate with poor clinical outcome. Future studies will further investigate the potential use of DUSP2 as a biomarker for lapatinib-resistance.

In summary, we have shown that hypoxia promotes lapatinib-resistance in ERBB2-positive breast cancer cells via HIF1-regulation of the ERK pathway. Our data also suggests that breast cancers containing high levels of HIF-1 and reduced DUSP2 expression could be utilized as predictive markers for lapatinib-resistance and identify patient population that may benefit from combination treatment with lapatinib and MEK inhibitors for the treatment of hypoxic ERBB2-positive tumors.

## MATERIALS AND METHODS

### Cell culture and reagents

MCF10A and SK-BR3 cells were obtained from ATCC (Manassas, VA). MCF10A-ERBB2 cells line is stably expressing wildtype ERBB2 (pBabe-Neu) as previously described [23]. MCF10A and MCF10A-ERBB2 cells were cultured in DMEM/F12 medium (Invitrogen, Carlsbad, CA) supplemented with 5% horse serum 20 ng/ml of EGF (Peprotech, Rocky Hill, NJ), 10 μg/ml of insulin (Sigma), 1 ng/ml of cholera toxin (Sigma), 100 μg/ml of hydrocortisone (Sigma), 50 units/ml of penicillin, and 50 μg/ml of streptomycin (Invitrogen); SK-BR3 in McCoy's medium (Invitrogen, Carlsbad, CA) supplemented with 10% fetal bovine serum. MTEC-Neu cells were kind gift from Tiffany Seagroves (University of Tennessee Health Sciences Center) and previously described [21]. LY294002 and U0126 were obtained from Calbiochem (San Diego, CA). Trametinib, lapatinib and dasatinib were purchased from Selleck Chemicals, LLC (Houston, TX).

### Hypoxic treatment

Cells were placed into a humidified hypoxic chamber (invivo2 Hypoxia Workstation, Ruskinn, UK) equilibrated to 1.0% O2, 5.0% CO2 at 37°C. Any experimental chemical compounds were added to cells immediately before placement in the hypoxic chamber. Cell lysates were collected immediately after removal from hypoxia chamber. Unless noted, all hypoxic treatments were performed for 48 hours.

### Growth inhibition assays

For all assays, unless noted, cells were cultured for 24 hours and then exposed to various concentrations of different chemical compounds for 48 hours. Cell viability was measured by colorimetric MTS assay Promega (Madison, WI) accordingly to manufacture instructions. Absorbance values were expressed as a percentage relative to untreated cells. Each sample was assayed in duplicate and each experiment was carried out three independent times. 0.1% crystal violet solution was used for crystal violet staining.

### shRNA transfections and viral transductions

Retroviruses were packaged as previously described [43] and used to stably transduce MCF10A-ERBB2 cells with pMiT-HIF-1α-P402A, P546A (kindly provided by D. Plas, University of Cincinnati) [44] and pLV158-DUSP2-flag (Genecopoeia, Rockville, PA). For shRNA transfections shRNA lentiviral particles were generated as previously describe [43]. Control pLKO-Puro vector containing shRNA from Addgene (plasmid 1864, D. Sabatini (MIT)) with sequence: cctaaggttaagtcgccctcgctcgagcgagggcgacttaaccttagg. HIF-1α and DUSP2 shRNA from Sigma and the sequences used for HIF-1α: ccggggagatcttgccctacctgttctcgagaacaggta gggcaagatctcctttttg and for DUSP2: ccggccgctggagacaca atcatatctcgagatatgattgtgtctccagcggtttttg.

### 3D morphogenesis assay and immunofluorescence

Assays were performed as described previously [20]. Briefly, 5000 cells per well were plated onto 8-well chamber slides (BD Falcon) coated with 40 μl of growth factor-reduced Matrigel (BD Falcon). Cells were then overlaid with growth medium supplemented with 2% growth factor-reduced Matrigel. Structures were allowed to form for six days and followed by drug treatments for 48 hrs. Immunofluorescence of three-dimensional structures was performed as previously described [20]. For quantification of percent cleaved caspase-3 positive acini; a minimum of 20 acinar structures were counted per experiment and each experiment was repeated three independent times. Caspase positivity was defined as a structure with 5 or more cleaved caspase-3-positive cells.

### Quantitative RT-PCR (qRT-PCR)

Total RNA was isolated from cells using the RNeasy Mini Kit (Qiagen, Hilden, Germany). Equal amounts of total RNA (250 ng) were added to Brilliant II qRT-PCR master mix (Stratagene, La Jolla, CA) with primer/probe sets purchased from Applied Biosystems (Foster City, CA). PCR were performed using a Applied Biosystems 7500 machine and analysis was performed using Data Assist (Stratagene, La Jolla, CA). Gene and catalog numbers for the primer/probe sets are as follows: DUSP2 (Hs00358879_m1). Expression of cyclophillin A (Hs99999904_ m1) and 18S (Hs99999901_s1) mRNA were as used as internal controls. Data are represented as a fold-change between samples.

### Immunoblot analysis

Cell lysates were prepared in RIPA lysis buffer supplemented with 1 μg each of pepstatin, leupeptin, aprotinin, and 200 μg/ml phenylmethylsulfonyl fluoride. Antibodies used include: human ERBB2, ERBB2 (pY1248), AKT, AKT (pS473), BIM, cleaved-caspase-3, p27, SRC, SRC(pY416), (Cell Signaling Technology Danvers, MA); HIF-1α were from Novus (Littleton, CO); β-actin, Erk1/2, pERK1/2, DUSP2 and HRP-conjugated secondary antibodies from Santa Cruz Biotechnology (Santa Cruz, CA).

### Clinical dataset analysis

The level of DUSP2 and HIF-1α mRNA expression in clinical breast tumor subtype from ER-negative breast cancers was derived from gene expression data and relapse free survival information was downloaded from GEO, EGA and TCGA database. For relapse-free survival analysis, we stratified ER-negative patients (*n* = 788) by expression of either DUSP2 (Affymetrix-ID- 204794) or HIF-1α (Affymetrix-ID-200989) and presented this data as Kaplan-Meier plots. Significance was determined using log-rank tests. (http:kmplot.com/analysis/index.php?p) [34].

### Statistical analysis

Quantitative data from all experiments are presented as means±s.e. from three independent experiments, and were analyzed with the unpaired two-tailed Student's *t*-test. A *P*-value of < 0.05 was considered statistically significant.

## SUPPLEMENTARY FIGURES


