# Supplementary material for: Hypoxia/HIF1α induces lapatinib resistance in ERBB2-positive breast cancer cells via regulation of DUSP2
Source: Oncotarget. 2015 Jan 24;6(4):1967–80. doi: 10.18632/oncotarget.2806 (PMC4385829; doi:10.18632/oncotarget.2806)
Supplement: Supplementary file 1 [file oncotarget-06-1967-s001.pdf]

SUPPLEMENTARY FIGURES

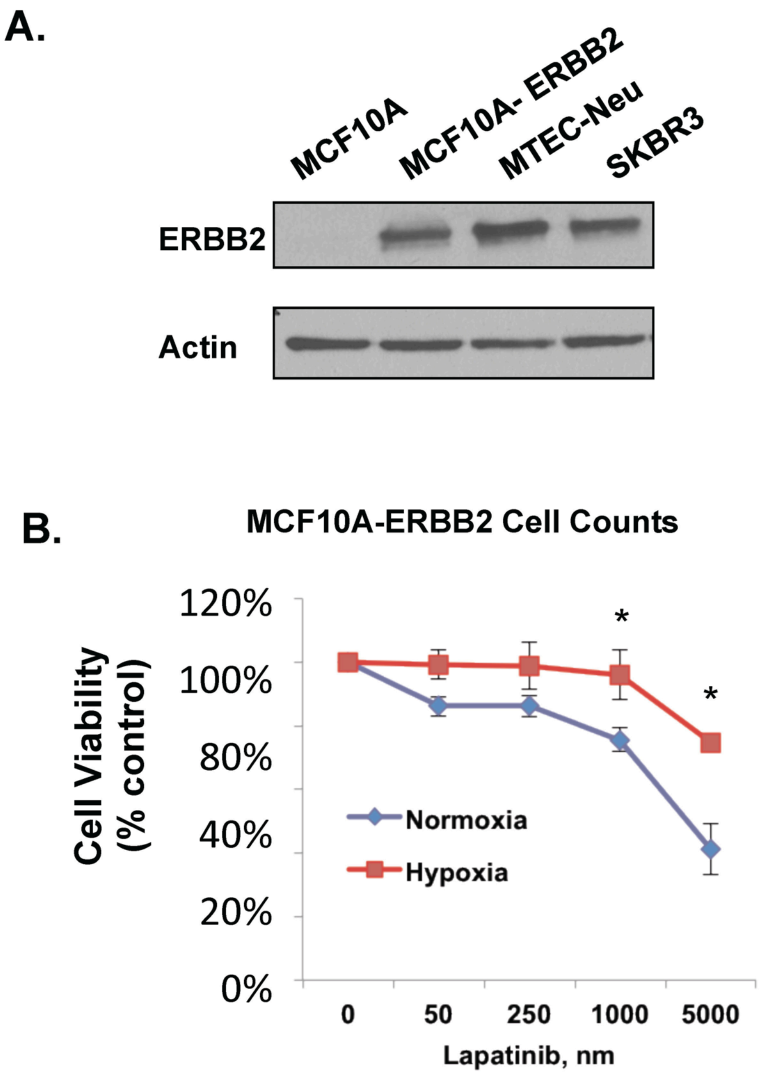

Supplementary Figure S1: Hypoxia blocks lapatinib-mediated growth inhibition in ERBB2-positive breast cancer cells. **(A)** ERBB2 expression was examined in a panel of cell lines via western blot analysis. **(B)** MCF10A-ERBB2 cells were treated with increasing doses of lapatinib under normoxic and hypoxic conditions. After 48 h treatment cell viability was assayed by cell counting. S.E. for $n = 3$. $*p \leq 0.05$.

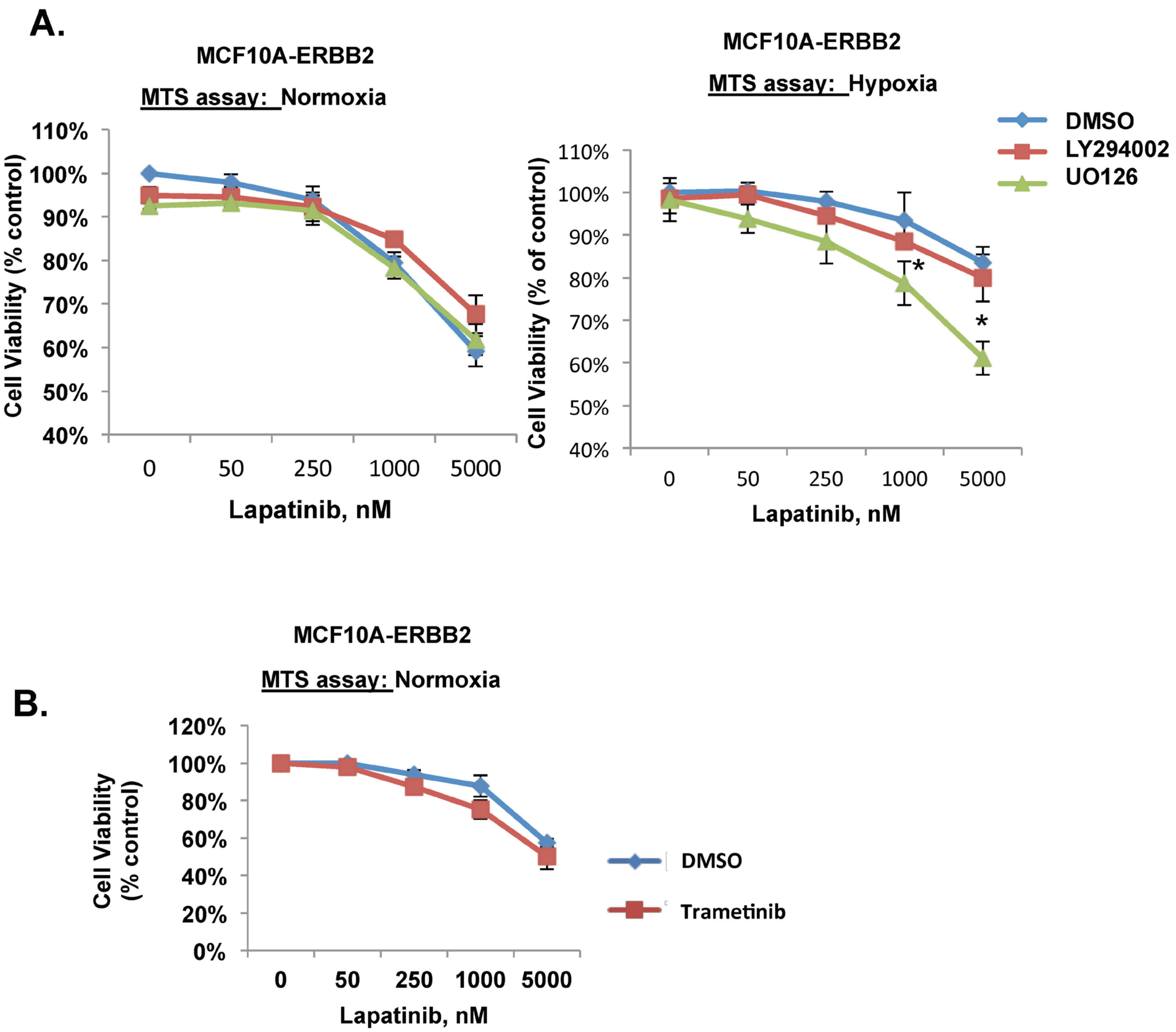

Supplementary Figure S2: Hypoxia requires ERK activity for resistance to lapatinib effects on breast cancer cells. **(A)** MCF10A-ERBB2 cells were treated with either MEK inhibitor (UO126, 5 µM), PI3-K inhibitor (Ly294002, 2.5 µM) under hypoxic conditions in presence of lapatinib. After 48 h treatment cell viability was assayed by MTS assay. **(B)** MCF10A-ERBB2 cells were treated with trametinib (50 nM) under normoxic conditions in presence of lapatinib. After 48 h treatment cell viability was assayed by MTS assay Error bars indicate S.E. for $n = 3$. $*p \leq 0.05$.

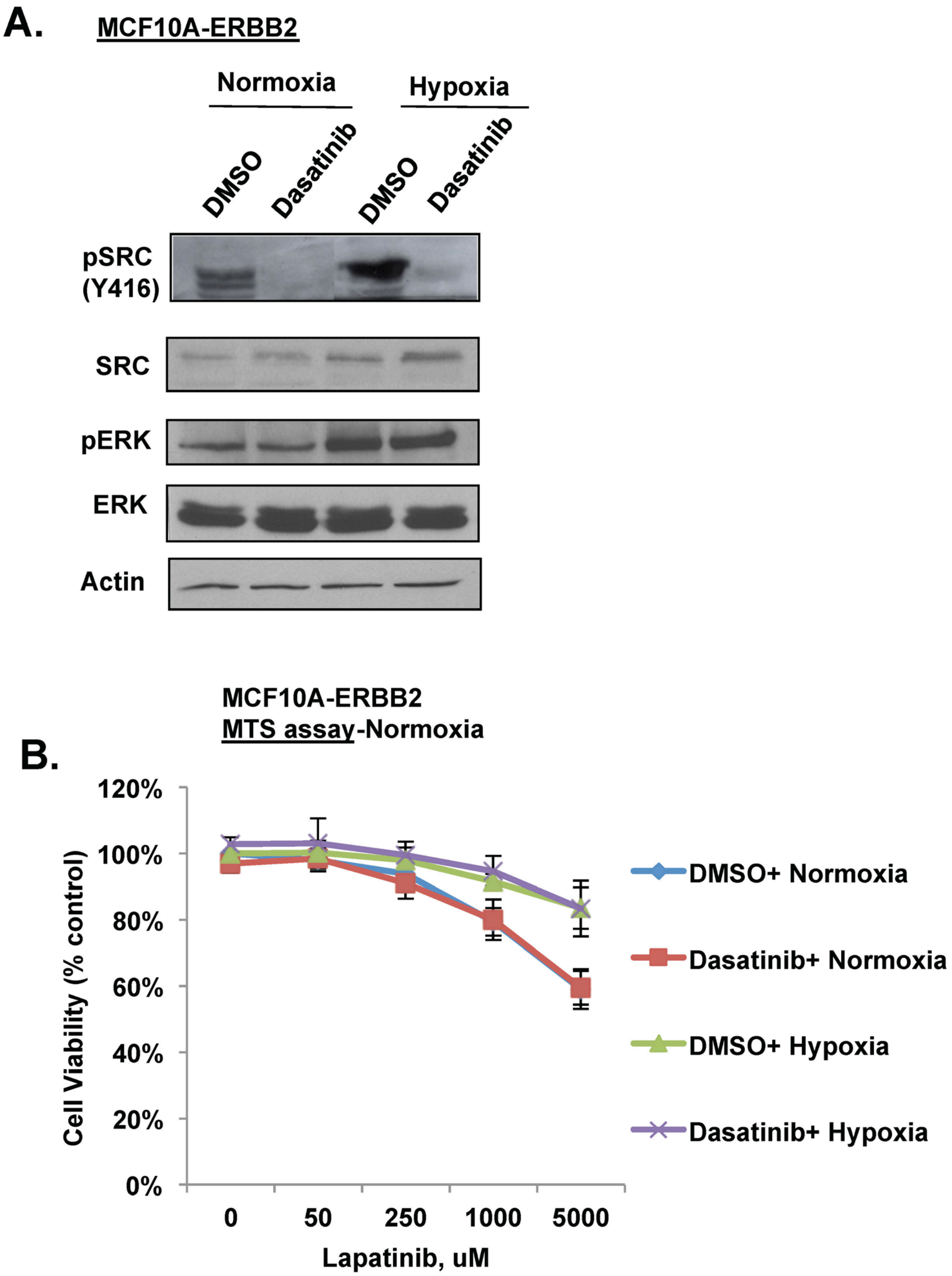

Supplementary Figure S3: c-SRC activation is not required for hypoxia-mediated lapatanib resistance and ERK activation. **(A)** c-SRC and ERK signaling was assayed via western blot analysis in MCF10A-ERBB2 cells treated with dasatinib (50 nM) under hypoxic and normoxic conditions. **(B)** MCF10A-ERBB2 cells were treated with dasatinib (50 nM) under hypoxic and normoxic conditions in presence of lapatinib. After 48 h treatment cell viability was assayed by MTS assay. Error bars indicate S.E. for $n = 3$.

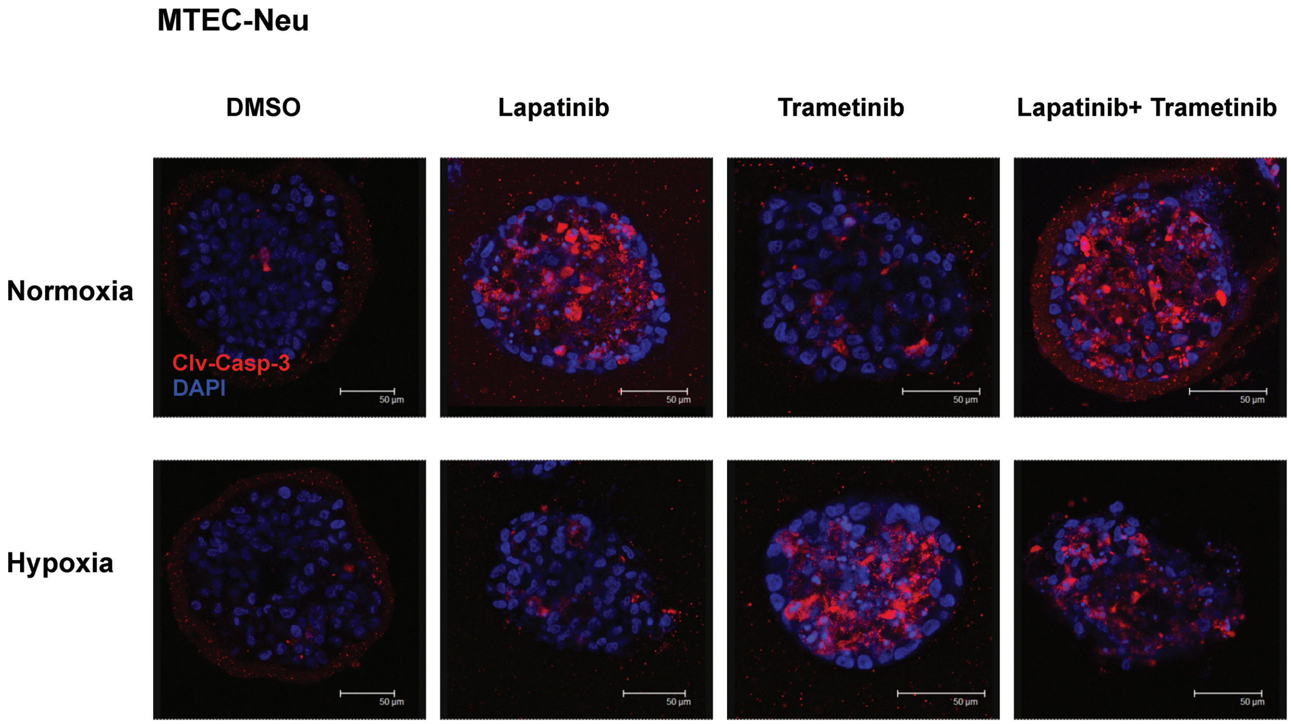

Supplementary Figure S4: Combination treatment of lapatinib and trametinib reverses hypoxic-mediated effects in MTEC-Neu cells. MTEC-Neu cells were cultured in 3D for 6 days and then treated with control, lapatinib (1 µM), trametinib (50 nM) alone or in combination under normoxia or hypoxia for 48 h. After 48 hours cells were fixed and stained for cleaved caspase-3/DAPI.

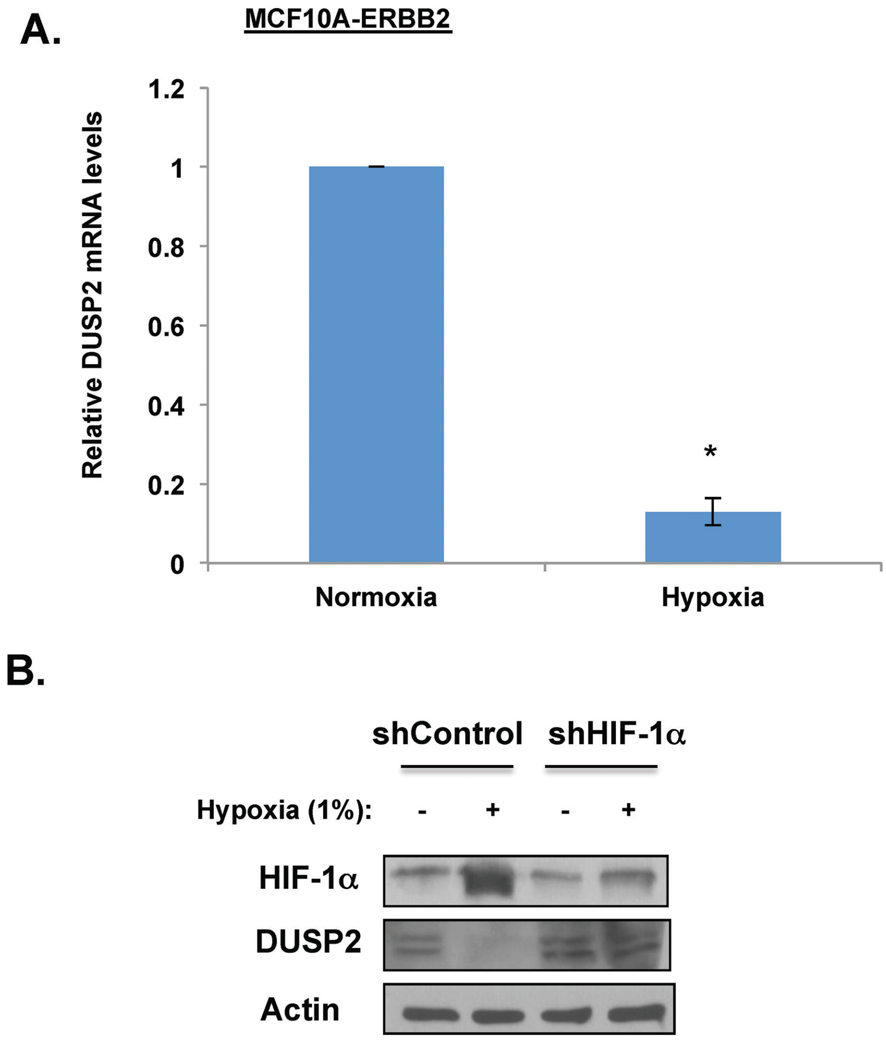

Supplementary Figure S5: Hypoxia decreases DUSP2 mRNA levels. **(A)** DUSP2 mRNA levels were measured in MCF10A-ERBB2 cells treated with hypoxia for 6 hours. **(B)** HIF-1α depleted MCF10A-ERBB2 cells were treated with hypoxia for 6 hours and DUSP2 and pERK levels were assayed via western blot analysis. Error bars indicate S.E. for $n = 3$, $*p \leq 0.05$.

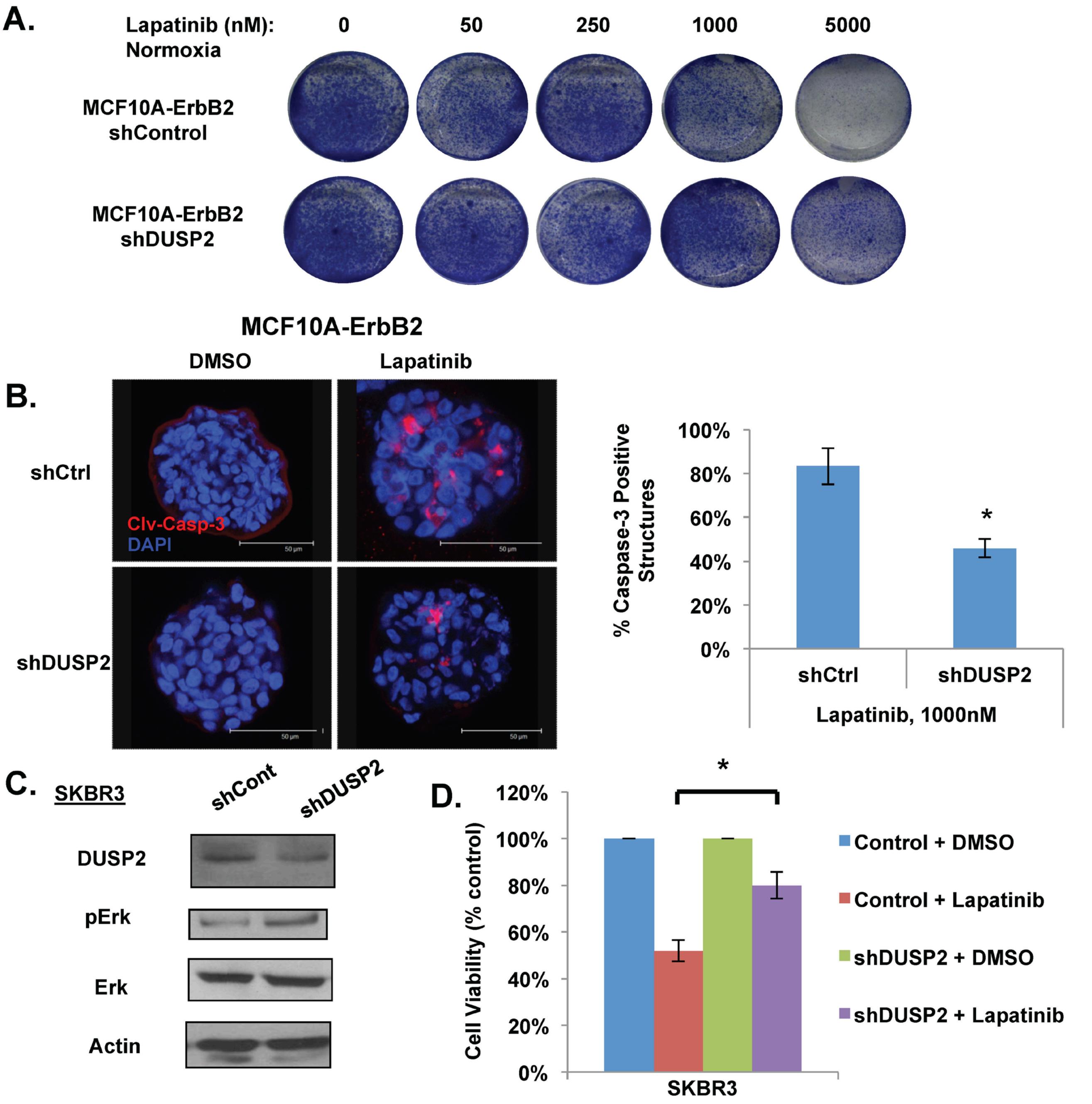

Supplementary Figure S6: HIF-1 target DUSP2 is required for hypoxia-mediated lapatinib resistance. (A) Cells expressing control or DUSP2 shRNA were placed in 3D culture conditions and then incubated under normoxic conditions in the presence or absence of lapatinib. Cells were stained for cleaved caspase-3 (top) and the percentage of caspase-positive acini was determined (right, bar graph). **(B)** Cells expressing control or DUSP2 shRNA under normoxia were treated with increasing doses of lapatinib and were stained with crystal violet. **(C)** DUSP2 levels and Erk activation was analyzed via western blotting in SKBR3 cells stably expressing control or DUSP2 shRNA. **(D)** SKBR3 cells stably expressing control or DUSP2 shRNA were treated with lapatinib under normoxic conditions. After 48 h treatment cell viability was assayed by MTS assay. Error bars indicate S.E. (*$p \leq 0.05$).

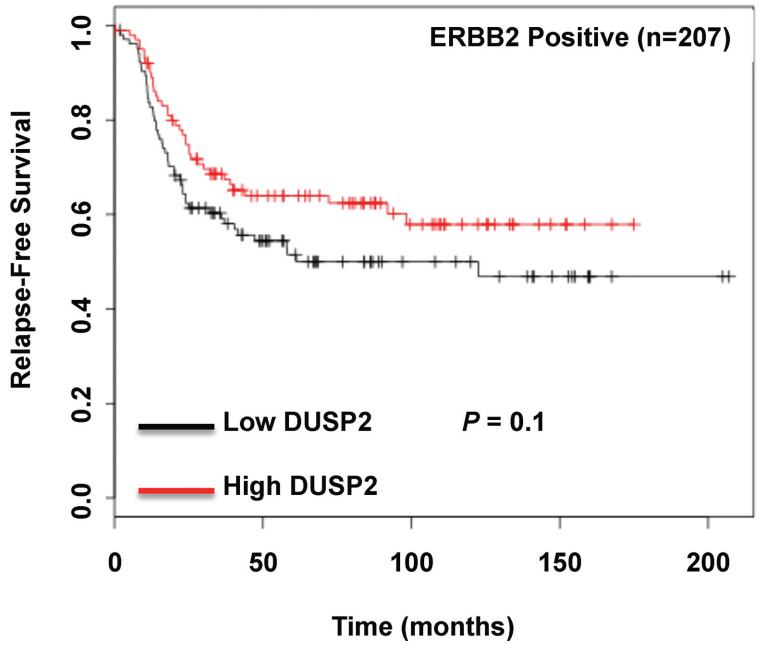

Supplementary Figure S7: Low DUSP2 level in ERBB2 positive breast cancer associates with decreased relapse-free survival. Kaplan-Meier plots of relapse-free survival in dataset of patients with ERBB2-positive breast cancer (*n* = 207), stratified by DUSP2 expression. Data was obtained from the Kaplan-Meier plotter database (Györffy et al., 2010). The *P* value was calculated by a log-rank test.
